# Clinicopathological and Radiological Features with Long Term Follow Up of Metaplastic Carcinoma Breast in India

**DOI:** 10.31557/APJCP.2021.22.11.3483

**Published:** 2021-11

**Authors:** Gopal Puri, Kush Raj Lohani, Sarada Khadka, Kamal Kataria, Piyush Ranjan, Smriti Hari, Sandeep Mathur, Anita Dhar, Anurag Srivastava

**Affiliations:** 1 *Department of Surgical Disciplines, All India Institute of Medical Sciences, New Delhi, India. *; 2 *Department of Radiodiagnosis, All India Institute of Medical Sciences, New Delhi, India. *; 3 *Department of Pathology, All India Institute of Medical Sciences, New Delhi, India. *

**Keywords:** Metaplastic breast carcinoma, triple negative breast cancer, overall and disease, free survival

## Abstract

**Objective:**

Metaplastic breast carcinoma (MBC) is a heterogeneous group of invasive carcinomas with squamous and/or mesenchymal differentiation. Because of their rare occurrence, the information regarding the clinical behaviour of metaplastic carcinomas is limited. The purpose of our study was to delineate the clinicopathological and radiological features, treatment outcomes, prognostic factors, and survival of patients with MBC.

**Methods:**

Ambispective observational study with prospective recruitment was done from 1^st^ January 2019 to 31^st^ August 2020. Retrospective data included between 1^st^ January 2009 and 31^st^ December 2018. In the retrospective group surgical database of our department was searched and those with MBC diagnosis on post-operative histopathology recruited. In prospective group patients with MBC on core biopsy were followed and those operated were included. The patients followed up at our breast cancer clinic (BCC) and their demographic, clinical, pathological radiological and treatment details noted.

**Results:**

Forty patients formed the study population. The mean age of the patents was 42 years. Ipsilateral axillary lymph node metastasis was present in 22.5%. The pathological median tumor size was 5.4 (range 2.1 to 22 cm). The most common differentiation was cartilaginous (35%) followed by squamous (32.5%). The most common mammographic grading was BIRADS 4 (Breast Imaging Reporting and Data system). Magnetic resonance imaging was T2 hyperintense with peripheral rim enhancement and restriction on DWI. The median overall (OS) and disease-free survival (DFS) was 42 and 40 months, respectively. Fifteen patients (37.5%) had disease related mortality. A subgroup analysis revealed that, type of differentiation, histopathology and tumor size > 5cm affected both OS and DFS significantly.

**Conclusion:**

Metaplastic breast cancer in our setup presents in young patients with aggressive large tumors at a higher stage and diverse histopathology and with comparable overall and disease-free survival. The histological subtype, tumor differentiation and tumor size are prognostic factors.

## Introduction

Traditionally the incidence of Metaplastic Breast Carcinoma (MBC) is 0.2%-5% of all invasive breast cancers (Lakhani et al., 2012). However, the recent studies have reported the incidence to be between 0.5%-2.2% (Hashmi et al., 2018; Moorman et al., 2020). Metaplastic breast cancer is a diverse group of invasive carcinomas with squamous with or without mesenchymal differentiation. They have an aggressive course. Based on histological characters, MBC has been classified into seven main types: matrix-producing/mesenchymal carcinoma, cartilaginous, spindle cell carcinoma, squamous cell carcinoma, osseous, sarcomatoid and adenosquamous (Lakhani et al., 2012). The immunohistochemical studies reveal primarily ER, PR negative and HER2/neu negative, but this is not absolute. There are other non-conventional immunohistochemical markers like cytokeratin, vimentin, S100, SMA4, BCL2, CD34 and P63

The clinical presentation of these cancers, in hindsight, reveal aggressively fast-growing, large tumors with paradoxically less rates of axillary metastasis at diagnosis (Alam et al., 2003; Lai et al.,2013). They are notorious to metastasise to the lungs among other organs. They portend a grave prognosis. Due to the lack of data, there are no clear guidelines on the specific management of these tumors and they are generally managed as the usual Invasive Ductal Carcinomas (IDC). Surgical management is the mainstay. Either breast conservation surgery (BCS) or mastectomy is done based on the tumor size and the breast tissue. The traditional chemotherapy drugs used in IDC have not shown much benefit in the MBC. Role of hormonal therapy is minimal with no role of HE2/neu inhibitors as most of these are triple negative breast cancers (TNBC) (Rayson et al., 1999; Aydiner et al.,2015). Radiation was earlier used only in the adjuvant setting (Pitts et al., 1991). Now recently conducted studies have shown that long term outcomes improved with neo-adjuvant chemotherapy and combined chemotherapy and radiotherapy (Li et al., 2019; Wang et al., 2019). The immunological studies have demonstrated a potential role of immunotherapy as it has high levels of PD-L1 and tumor infilterating T lymphocytes (Gadaleta-Caldarola et al., 2021).

Because of the rare occurrence of MBC, the information regarding the clinical behaviour and tumor biology of metaplastic carcinomas is scarce and is primarily based on small retrospective studies. Hence, the present study was designed to evaluate overall survival, disease free survival, clinical outcomes and prognostic factors of patients with histologically diagnosed MBC.

## Materials and Methods

*Study Design*

An ambispective observational study was conducted in the department of surgical disciplines at the All India Institute of Medical Sciences, New Delhi, India with prospective recruitment done from 1st January 2019 to 31^st^ August 2020. Retrospective data included patients operated in between the period of 1st January 2009 to 31^st^ December 2018. 

*Methodology*

The surgical database of our department was searched for the term metaplastic carcinoma and the patients with confirmed post-operative histopathological report were recruited in the retrospective group. These patients were under regular follow up at our breast cancer clinic (BCC). The demographic, clinical, pathological radiological and treatment details of the patients were noted in their BCC file. They were called for follow up regularly to the BCC at an interval of 3 months for the first 2 years, then 6 monthly for another 3 years and annually thereafter. The BCC file was updated at each visit. During the COVID-19 pandemic, the follow up was done via teleconsultations involving voice and video calls.

*Inclusion and Exclusion Criteria*

For the retrospective group patients with a confirmed of metaplastic carcinoma on the histopathology of the surgically excised specimen were included. Only those which were operated between the recruitment period were included. The patients which not operated (due to various reasons), but MBC was suspected on the core biopsy were excluded from recruitment. Similarly in the prospective group, all the patients with suspected diagnosis on a core biopsy followed up and only the ones operated were included after the diagnosis was confirmed by the operative specimen histopathology.

*Statistical Analysis*

Mean of variables was calculated when the data was evenly distributed and median when the variable was skewed. Survival analysis was done using Kaplan-Meier curves using sigma plot 12.3 software. The overall (OS) and Disease-free survival (DFS) was calculated and compared for the subgroup analysis to delineate the prognostic factors. A study from the western population was selected with a similar design and sample size for comparison (El Zein et al., 2017)

## Results

A total of 51 patients were considered for recruitment. Forty-four patients were in the retrospective group out of which 3 were excluded due to metastasis at diagnosis and hence, were not operated. Six patients (13.04%) were lost to follow up so excluded from the final analysis. Out of the 7 patients in the prospective group, 2 were excluded as they were receiving NACT at the conclusion of recruitment period and were not operated. Thus, a total of 40 cases were included for further analysis (35 retrospective and 5 prospective). Due to small numbers, only a combined analysis was performed. 

*Clinical Features*

The mean age of 40 patients was 42 years with standard deviation of 12 years. Twenty-two (55%) patients were pre-menopausal at the time of diagnosis. Twenty-three patients (57.5%) had a T3 tumor and 9 (22.5%) patients had metastasis to the ipsilateral axillary lymph nodes. Fifteen (37.5%) patients were Stage III at presentation and 17 (42.5%) had received neo-adjuvant chemotherapy. Twenty-seven (67.5%) patients underwent mastectomy and breast conservation surgery could only be done in remaining 13 (32.5%) ladies. Table 1 delineates the details of clinical and pathological features.

*Pathological Features*

**Figure 1:**
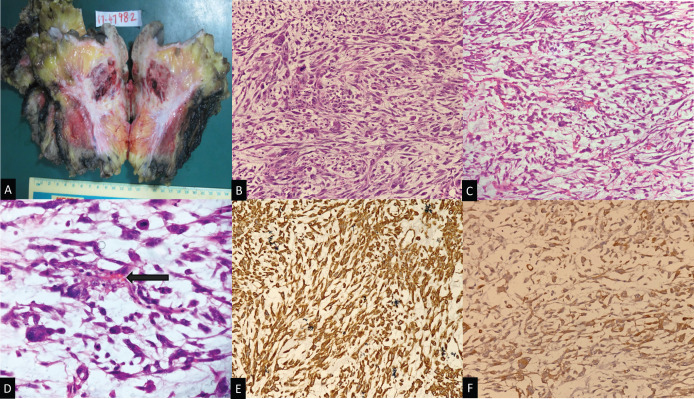
Gross and Histopathological Images of Metaplastic Bbreast Carcinoma(MBC). A, Gross specimen with scirrhous centre depicting the mesenchymal differentiation; B, HPE 20X depicting Cartilagenous variant of MBC; C, HPE 20X depicting Squamous variant of MBC; D, HPE 40X with marked arrow at the vascular invasion by the tumour emboli; E, Immunohistochemical marker Cytokertain-stained MBC; F, Immunohistochemical marker Vimentin-stained MBC

On gross examination, these tumors were scirrhous with regular or irregular margins (Figure 1A). The pathological tumor size ranged from 2.1 cm to a whopping 22 cm with a median tumor size of 5.4 cm. The most common differentiation was cartilaginous in 14 (35%) followed by squamous (32.5%) (Figure 1B, 1C). Twelve (30%) tumors had more than one type of differentiation with the rest having a pure metaplastic component. There was lympho-vascular invasion in 18 (45%) cases and is depicted in Figure 1D. Necrosis was seen in 32(80%) tumors. Only 2 cases were ER and PR positive with Her2neu negative. There were a variety of immunohistochemical markers seen, with cytokeratin (50%) and vimentin (35%) (Figure 1E, 1F) being the most common (Table 1). 

*Radiological Features*

**Figure 2:**
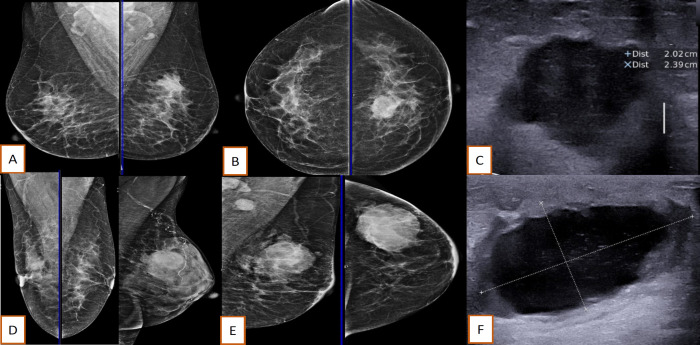
Mammo-sonographic images of Metaplastic Breast Carcinoma(MBC). A & B, Irregularly shaped high-density mass with indistinct margins (BIRADS 4c) in a mammogram of a 47 year old lady; C, Ultrasonography of the same lady depicting the mass to be hypoechoic with micro-lobated margins; D, Mammogram of two ladies showing iso-dense oval mass with pleomorphic calcification (BIRADS 4c); E, Mammogram of 41 year old lady with oval high-density circumscribed mass with partly indistinct margins with skin thickening and axillary nodes (BIRADS 4b); F, Ultrasonography of the same lady with complex cystic lesion

**Figure 3:**
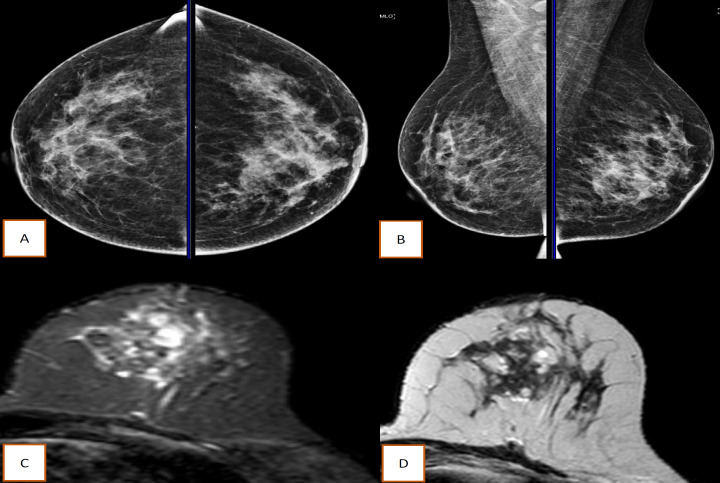
Mammographic and MRI features of MBC. A & B, Focal asymmetry in the left breast without any lump on mammography; C, MRI of the left breast showing T2 hyperintensity; D, Same lesion showing peripheral enhancement with surrounding non-mass clustered rim enhancement on the diffusion weighted imaging sequence of MRI

The radiological features were noted in only 22 patients whose investigations were done at our centre. Metaplastic breast cancer on ultrasonography showed diverse range of findings from being a complex cystic lesion to a hypoechoic mass with micro-lobation (Figure 2C, 2F). The mammographic findings are summarised in Table 2. American College of Radiology grade D breast density was seen in 9 (40.1%) cases and BIRADS 4 (Figure 2) was the most common mammographic grading reported. Magnetic resonance imaging was done for the 2 cases with only focal asymmetry without any mass lesion. The core biopsy was taken by ultrasonographic guidance in these cases. The MBC was heterogenous T2 hyperintense with peripheral rim enhancement and restriction on diffusion weighted imaging (Figure 3).

*Clinical Outcomes and Survival*

**Figure 4:**
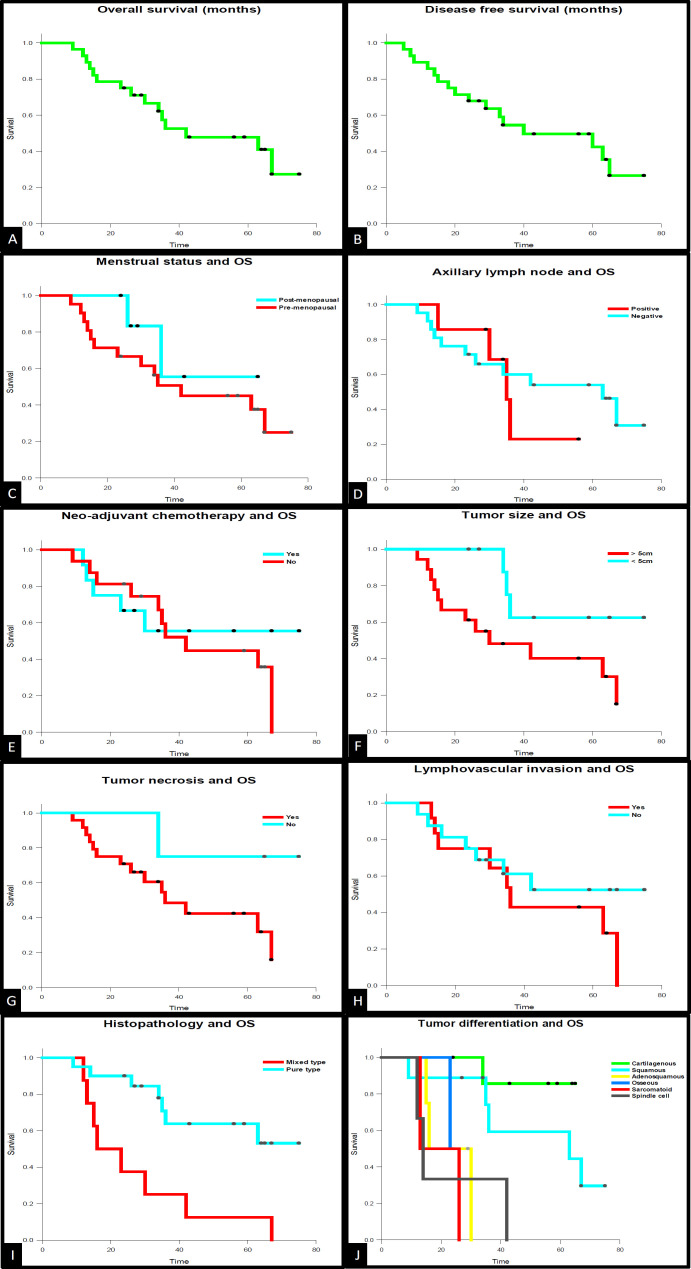
Kaplan-Meier Survival curves. A, Overall survival (OS) of our study cohort; B, Disease-Free Survival (DFS) of our study cohort; C-J, Subgroup Kaplan-Meyer survival analysis of Overall survival with various factors as mentioned. Tissue differentiation, MBC histology and tumor size> 5cm affected the OS and DFS significantly. Whereas tumour necrosis, lympho-vascular invasion and axillary lymph node involvement showed stark difference in the survival months but this was not statiscally significant

At the end of the study period (last follow up visit on 30th January 2021) 18(45%) were alive and disease free, 5 (12.5%) were alive with the disease and there were 17 (42.5%) mortalities. Out of these, 2 (11.7%) mortalities were from non-disease related causes. Of the 15 (88.23%) disease related mortalities, lung metastasis was present in 8 (53.33%) cases, metastasis to other organs was seen in 5 (33.33%) patients and 2 (13.34%) had loco-regional recurrence with intrathoracic extension without distant metastasis. Out of the 40 patients the follow up period of 10 patients was less than 2 years, so they were excluded from the survival analysis. Apart from this, the 2 patients which died of non-disease related conditions (acute myocardial infarction) are also excluded. Hence, the survival analysis is done for 28 patients. The median overall (OS) and disease-free survival (DFS) was 42 and 40 months, respectively. A subgroup analysis of OS and DFS is depicted in table 3. It revealed that, type of differentiation, histopathology and tumor size > 5 cm affected both OS and DFS significantly. Axillary lymph node involvement reduced OS from 63 months to 35 months (Table 3). Other prognostic factors such as menopausal status, tumor necrosis and lympho-vascular invasion did not affect survival. The Kaplan-Meier curves of these analysis are depicted in Figure 4.

**Table 1:** Clinico-Pathological Findings of the Patients of Metaplastic Breast Cancer(n=40)

Clinicopathological Parameters		Value
Age (Years)	Mean	42.10
	SD	12.19
Menopausal Status	Pre-Menopausal	22 (55%)
	Post-Menopausal	18 (45%)
Tumour Staging	T2	17 (42.5%)
	T3	23 (57.5%)
Nodal Staging	N1	6 (15%)
	N2	2 (5%)
	N3	1 (2.5%)
TNM Staging	Stage II	25 (62.5%)
	Stage III	15 (37.5%)
NACT Given		17 (42.5%)
Hormonal Status	Triple Negative	38 (95%)
	ER PR + Her2neu -	2 (5%)
Surgery Performed	WLE plus SLNB +/- ALND	13 (32.5%)
	Simple mastectomy Plus SLNB	13 (32.5%)
	MRM	14 (35%)
Pathological Tumour size(cm)	Median	5.40
	Mean with SD	6.5 +/- 4.6
	Range	2.1 to 22
Histopathological Hallmarks	Necrosis	32 (80%)
	Lympho-vascular Invasion	18 (45%)
Predominant type of differentiation	Cartilaginous	14 (35%)
	Squamous	13 (32.5%)
	Adeno-squamous	4 (10%)
	Osseous	3 (7.5%)
	Sarcomatoid	2 (5%)
	Spindle cell	4 (10%)
Histology	Pure metaplastic	28 (70%)
	Mixed metaplastic	12 (30%)
IHC markers	Cytokeratin	20 (50%)
(Multiple positive can be present)	Vimentin	14 (35%)
	S-100	12 (30%)
	SMA	6 (15%)
	BCL-2	4 (10%)
	CD 34	2 (5%)
	P63	4 (10%)
Clinical Outcomes	Alive and disease free	18 (45%)
	Alive with local recurrence	2 (5%)
	Alive with Distant metastasis	3 (7.5%)
	Mortality	17 (42.5%)

ALND, Axillary Lymph Node Dissection; BCL-2, B cell CLL/lymphoma-2; CD, Cluster of differentiation; MRM, Modified Radical Mastectomy; SD, Standard Deviation; SLNB, Sentinel Lymph Node Biospy; SMA, Smooth muscle antigen; WLE, Wide Local Excision.

**Table 2:** Mammo-Sonographic Findings of Metaplastic Breast Carcinoma

Radiological Parameters		No. of Patients (n=22)
ACR Category of Breast Density	A	0
	B	6 (27.27%)
	C	7 (31.81%)
	D	9 (40.10%)
BIRADS Classification (On Mammo-sonography)	Category 3	2 (9.09%)
	Category 4	10 (45.45%)
	Category 5	7 (31.81%)
	Category 6	3 (13.63%)
Abnormal Findings	Mass lesion	20 (90.90%)
	Focal asymmetry	2 (9.09%)
Margins of the lesion	Spiculated	0
	Partially circumscribed	9 (40.10%)
Shape of the mass lesion	Oval	12 (54.54%)
	Irregular	8 (36.36%)
Miscellaneous features	Suspicious Nodes	4 (18.18%)
	Pleomorphic Calcification	3 (13.63%)

ACR, American College of Radiology.

**Table 3:** Survival Analysis and the Subgroup Analysis of the Prognostic Factors in MBC (n=28)

Group	Overall survival (months)	P value	Disease free survival (months)	P value
	[95% Confidence Interval)		[95 % Confidence Interval)	
Survival of the Cohort	42 (7.3 – 76.6)	N.A	40 (7.5 – 72.4)	N.A
Menstrual status				
Post-menopausal (n=7)	Not Reached	0.35	Not Reached	0.26
Pre-menopausal (n=21)	42 (7.8 – 76.1)		40 (3.2 – 76.7)	
Axillary lymph node				
Positive (n=7)	35 (29.2 – 40.7)	0.63	33 (24.7 – 41.2)	0.62
Negative (n=21)	63 (35.9 – 90)		60 (28 – 91.9)	
Neo-adjuvant chemotherapy	Not Reached			
Yes (n=12)	42 (29.7 – 54.2)	0.61	65 (4.8 – 125.1)	0.9
No (n=16)			40 (10.9 – 69)	
Tumor size				
> 5cm (n=18)	30 (8.3 – 51.6)	0.06	24 (-9.2 – 57.2)	0.02
< 5cm (n=10)	Not Reached		Not Reached	
Tumor Necrosis				
Yes (n=24)	36 (21.3 – 50.6)	0.13	40 (11 – 68.9)	0.04
No (n=4)	Not Reached		Not Reached	
Tumor differentiation				
Cartilagenous (n=8)	Not Reached	0.002	Not Reached	<0.001
Squamous (n=9)	63 (-4.4 – 130.4)		60 (25.3 – 94.6)	
Adenosquamous n=4)	16 (6.2 – 25.8)		15 (5.2 – 24.8)	
Osseous (n=2)	23 (- inf to + inf)		20 (- inf to +inf)	
Sarcomatoid (n=2)	13 (-5 – 31)		5 (-7.4 – 17.4)	
Spindle cell (n=3)	14 (10.7 – 17.2)		12 (5.5 – 18.4)	
MBC Histopathology				
Mixed (n=8)	16 (4.9 – 27)	0.002	15 (6.6 – 23.3)	<0.001
Pure (n=20)	Not Reached		65 (26 – 103.9)	
Lymphovascular invasion				
Yes (n=12)	36 (27.3 – 44.6)	0.34	33 (21.9 – 44)	0.28
No (n=16)	Not Reached		65 (22.7 – 107.2)	

**Table 4:** Comparison of Outcomes with the Western Population in the Study by El Zein et al

Clinicopathological Parameters		Our Study	Study by El Zein et al
Age (Years)	Median	41.5	50
	Range	19 to 78	42 to 90
TNM Staging (n=40)	Stage I	0	12 (30%)
	Stage II	25(62.5%)	23 (57.5%)
	Stage III	15 (37.5%)	5 (12.5%)
NACT Given(n=40)	Yes	17 (42.5%)	9 (22.5%)
	No	23 (57.5%)	31 (77.5%)
Hormonal Status (n=40 ours n=46 for Zein et al)	Triple Negative	38 (95%)	37 (80.4%)
	ER	2	4
	PR	2	6
	Her 2 neu	0	3
Surgery Performed (n=40)	BCS	13 (32.5%)	17 (42.5%)
	Mastectomy	27 (67.5%)	23 (57.5%)
Pathological Tumour size(cm)	Median	5.4	3.1
	Range	2.1 to 22	0.5 to 14
Predominant type of differentiation (n=40 and n=46)	Cartilaginous	14	3
	Squamous	13	12
	Mesenchymal	0	17
	Spindle cell	4	4
Histology (n=40 and n=46)	Pure metaplastic	28 (70%)	9 (19.6%)
	Mixed metaplastic	12 (30%)	37 (80.4%)
Clinical Outcomes (n=40)	Alive and disease free	18 (45%)	15 (37.5%)
	Alive with local recurrence/metastasis	5 (12.5%)	13 (32.5%)
	Mortality	17 (42.5%)	12 (30%)

## Discussion

This study presents clinical features, outcomes, and prognostic factors of forty patients of MBC, managed at our tertiary care institute. Generally, MBC presents with bigger size, higher grade and stage, triple negative hormone receptors with less metastasis to the lymph nodes and more possibility of distant metastasis when compared with Invasive Ductal Carcinoma (IDC) (Lai et al.,2013; Vias et al.,2019). Patients with MBC usually present at a median age of around 60 years (Rayson et al., 1999) while earlier diagnosis at the mean age of 50 years is also reported (Pitts et al., 1991). However, our patient presented at a markedly younger age of 42 years. The axillary lymph nodes involvement was 22.5% in our cohort. There are huge discrepancies in the prevalence of the lymph node metastasis which ranges from 0-63% according to various studies (Pitts et al., 1991; Al Sayed et al., 2006; Leddy et al., 2012; Lee et al., 2012; Schwartz et al., 2013; Song et al., 2013; Zhang et al., 2015) but is considered to be around 26%. This is considerably lower than the percentage of involvement in the IDC (30-45%) (Song et al., 2013). 

Due to the rarity of this tumor, there are no standardised protocols for the management of this subtype (Gradishar et al., 2017). But given the poor response to chemotherapy, surgical excision is considered the primary modality of treatment (Corso et al., 2021). Majority of the cases underwent mastectomy owing to the large tumor size at presentation and BCS was done only in 32.5% of our patients. Recently published study done on the Surveillance, Epidemiology, and End Results (SEER) database concluded that BCS conferred better OS and breast cancer specific survival as compared to mastectomy and this was seen for all T stages and N stages, except for N2-3 disease (Zhang et al., 2021). Neoadjuvant chemotherapy was given to 42.5% cases, this is lower than the data reported in literature for MBC (53.4%) (Pezzi et al., 2007). However, the recent studies have reported up to 90% patients receiving chemotherapy in the form of neoadjuvant therapy and adjuvant therapy (Hennessy et al., 2006; Fayaz et al.,2017). Administration of combined chemotherapy and radiotherapy in cases with node positive cases has shown to prevent micro-metastasis and improve the survival outcomes (Ma et al., 2021).

The pathological features demonstrate the actual uniqueness of this type over the IDC. Metaplastic breast cancer is known for larger tumor sizes. The mean tumor size reported for IDC is 2.7cm as compared to 5cm for the metaplastic variant (Yu et al., 2015; El Zein et al., 2017; Donato et al., 2018). The recent studies report a median size of 2.2 cm even in MBC cases (Moorman et al., 2020). The median tumor size in our study was 5.4 cm ranging from 2.1 cm to 22 cm. However, there are other studies also which have reported unusually large tumors (Abbas-Zadeh, 1992). Metaplastic breast cancer has distinct types of differentiation and the types can co-exist. There are studies that has associated these subtypes with outcome. The matrix- producing tumors had the best outcome while the spindle, mixed spindle and squamous carcinomas had the worst outcome, and this was an independent prognostic variable (Beatty et al., 2006; Nayak et al., 2013; Tadros et al., 2021). In comparison to this, our study reported the best outcome with cartilaginous and worst with spindle cell and sarcomatoid. The most common differentiation in our set up was cartilaginous (35%) followed by squamous (32.5%). This is consistent with the most common subtype reported in literature, squamous carcinoma (40%), followed by matrix-producing/mesenchymal and cartilaginous MBC (Choi et al., 2012; Jia et al., 2019; Puii et al., 2020). However, there were no matrix producing tumor in our study. Almost all the patients had a triple negative tumor (95%) in our study, which was more than the reported percentage in the literature ranging between 70% and 85% (Leibl et al., 2005; Beatty et al., 2006; Tse et al., 2006; Casey et al., 2007; El Zein et al., 2017). There was no Her2/neu enriched tumor in our cohort, but the reported positivity is 0–25% in the literature (Ong et al., 2018). There are other IHC markers such as Cytokeratin, Vimentin, S-100, SMA, Bcl-2, p63 and CD34 which help to differentiate these tumors from their close differential diagnosis. These are primary sarcoma of breast, myoepithelial breast carcinoma, myofibroblastic tumors, phyllodes tumors, nodular fasciitis, fibromatosis, and pleomorphic adenoma (Arias-Stella et al., 2018). These are usually CD 34 negative as it is positive in phyllodes, and sarcoma of the breast. Although mixed tumors are rare, there are case reports of MBC with cartilaginous differentiation with a co-existing foci chondrosarcoma with lympho-vascular invasion (Vias et al.,2019).

Our study includes an extensive follow up of the patients and only six were lost to follow up out of 46 (13.04%). At the time of conclusion of study, 45% were alive and disease free, 12.5% were alive and disease free and there was 42.5% mortality, out of which 11.7% patients died of non-disease related causes. Out of those died due to disease related causes, 86.66% died of metastasis and 13.34% due to loco-regional reccurence. Overall metastasis was present in 40% cases and loco-regional recurrence in 10% patients. This is consistent with the literature reports of 5-12% of loco-regional recurrence and about 40% of metastatic disease (Luini et al., 2007; Song et al., 2013; Edenfield et al., 2017). The route of metastasis is by hematogenous invasion, resulting in bone and lung metastasis (Luini et al., 2007). The survival of the patients with MBC is decreased as compared to the IDC (Ong et al., 2018; Han et al., 2019). The overall survival (OS) in our cohort was 42 months, in accordance with the reported survival is 48-60 months (Fayaz et al., 2017; Pukkala et al., 2018). The largest series reported from India with 31 patients reported survival to be 39 months (Puii et al., 2020). Axillary lymph node involvement reduced the OS in our study, but this was not significant statistically. Post-menopausal patients had better survival, since this tumor is not associated with hormone receptors, this can be explained by the unfavourable biology of the tumor originating at an early age. Another interesting finding highlighted by our study was the decreased survival in those not receiving NACT, which might shed a light on the role of chemotherapy in this disease. There was no mortality in the group of 4 patients who did not have necrosis in their final histopathology and thus both OS and DFS for that group was not reached compared to OS and DFS 36 months and 40 months, respectively, in patients with presence of tumor necrosis. Similar finding was seen in case of lympho-vascular invasion of the tumor. However, both of these were not statistically significant. The survival outcome in our study was significantly affected by the tumor size more than 5 cm, type of differentiation and pure or mixed histology of the tumor. In a study at the Johns Hopkins, OS with tumors more than 5 cm was decreased as compared to those presented with tumors less than 2cm (Fayaz et al., 2017). It also confirms the poor prognosis of this disease with 5 years OS of only 54.8%, as compared with of 86%-88% in breast cancer as reported in the Nordic Cancer Registry between 2000 and 2014 (Pukkala et al., 2018). 

The findings of our study were contrasted to a study done in the western population with a comparable sample size (El Zein et al., 2017). It included 46 patients of MBC with a subgroup analysis done of 40 patients. A comparison was made, as shown in table 4. In their population, the median age was 50 years with 12(30%) presenting in stage I. The median tumor size was 3.1 cm with maximum tumor being 14 cm. Predominant histology was mesenchymal (17 cases) while pure MBC was noted only in 9 cases. The mortality was in 12 (30%) patients with recurrence and/or metastasis seen in 13 (32.5%) patients. Our patients presented at a much younger age, but also at later stage. Most of our patients were at stage II while 30% of the western population were detected in stage I. Moreover, the tumor size in our population is bigger (5.4 vs. 3.1 cm). This can be attributed to early detection and probably a less aggressive disease in the western population as also has been evident from lower mortality compared to ours (42.5% vs 30%). Few other studies have reported OS of 54-83% (Toumi et al., 2011; Abouharb et al., 2012; Esbah et al., 2012; Cimino-Mathews et al., 2016; Langlands er al., 2016; Jha et al., 2017) 

This is the first study of its kind from the Indian population on metaplastic breast cancer with extensive follow up and analysis of prognostic factors. To the best of our knowledge, this study reports the maximum number of cases from the country. The limitation of this study is the retrospective component with selection bias. The study was conducted in a single institute. The study was concluded in a short interval (5 months) after the last recruitment so 5-year survival rates could not be calculated.

Metaplastic breast cancer is a heterogeneous disease encompassing biologically different tumor classes with variable outcome. Metaplastic breast cancer in the Indian setup presents in younger patients with aggressive large tumors at a higher stage and diverse histopathology and with comparable overall and disease-free survival. Histological subtype, differentiation and tumor size are prognostic factors. There is a need for a multi-institutional prospective study with detailed pathological analysis with longer follow up period for identifying definitive prognostic and predictive factors for developing a standardised management regimen.

## Author Contribution Statement

We would like to acknowledge the contribution of the various authors, starting from author 1 in data collection and writing the manuscript, author 2 for the data analysis and manuscript writing, author 3 for the extensive clinical follow up, author 4 and 5 for editing the manuscript, author 6 to provide the radiological correlation, author 7 for the pathological outcomes and author 8 and 9 for conceptualisation and their constant guidance.

## Statement conflict of Interest

No conflict of interest.
